# Integrated Proteomic and Metabolomic Profiling of the Secretome of *Fusarium verticillioides* Reveals Candidate Associated Proteins and Secondary Metabolites

**DOI:** 10.3390/jof12010024

**Published:** 2025-12-27

**Authors:** Min-Min Sui, Yan Zhang, Jian-Fa Yang, Fan-Fan Shu, Feng-Cai Zou, Jun-Jun He, Jun Ma

**Affiliations:** 1 Faculty of Animal Science and Technology, Yunnan Agricultural University, Kunming 650201, China; smm9797@163.com (M.-M.S.); zynldd123@163.com (Y.Z.); jsc315@163.com (J.-F.Y.); shuff1227@163.com (F.-F.S.); zfc1207@vip.163.com (F.-C.Z.); 2 The Yunnan Key Laboratory of Veterinary Etiological Biology, College of Veterinary Medicine, Yunnan Agricultural University, Kunming 650201, China; 3 College of Animal Husbandry and Veterinary Medicine, Yunnan Agricultural and Vocational Technical College, Kunming 650201, China

**Keywords:** *Fusarium verticillioides*, fumonisins, secretions, proteomic, metabolomic

## Abstract

*Fusarium verticillioides* (*F. verticillioides*) is an important fungal pathogen known to infect a variety of economically critical crops, particularly maize, causing substantial yield reductions and economic losses worldwide. In addition to its direct damage to agricultural productivity, *F. verticillioides* threatens public health by producing/secreting potent compounds, including well-known fumonisins (FUMs), which pose significant health threats to both livestock and humans due to their toxicity and carcinogenicity. However, current knowledge of the materials secreted/produced by *F. verticillioides*, such as secreted proteins and additional secondary metabolites, remains limited. In the present study, we conducted an integrated secretome analysis of *F. verticillioides* at the exponential growth stage by using proteomic and metabolomic technologies. The results of the present study showed that proteomic analysis identified 185 proteins, including 138 fungus-specific proteins. GO enrichment of these 138 fungus-specific proteins yielded 24 significant terms spanning carbohydrate/polysaccharide and aminoglycan metabolic/catabolic processes, extracellular and membrane-anchored components, and hydrolase/peptidase activities. Meanwhile, KEGG analysis identified starch and sucrose metabolism as the sole significantly enriched pathway. Metabolomic analysis of medium supernatant showed that a total of 2352 metabolites were identified, with 110 unique to the medium supernatant of the fungal group, including fumonisins (A1, B2, B3, B4), fatty acids, and other bioactive compounds. KEGG pathway enrichment highlighted key metabolic pathways, including the TCA cycle, unsaturated fatty acid biosynthesis, and arachidonic acid metabolism. These findings provide new insights into the pathogenic mechanisms of *F. verticillioides*, suggesting candidates for virulence-associated functions and metabolic adaptations that potentially contribute to its pathogenicity.

## 1. Introduction

*F. verticillioides* is a globally distributed pathogen that poses severe threats to plants [1]. Epidemiological surveys have shown that *F. verticillioides* has been distributed in maize-growing regions worldwide. The prevalence of *F. verticillioides* in North America [2], Europe [3], Africa [4], and Asia [5] ranges from 60% to over 90%, illustrating its global and serious impacts on the agricultural industry [6]. For example, this fungus can induce a range of devastating diseases in maize, including root rot [7], stalk rot, seedling blight, and ear rot [8], resulting in significant yield losses and substantial economic impacts worldwide. Its pathogenicity is not limited to maize alone, but also extends to other crucial cereal crops, affecting key crops such as rice [9,10,11], wheat [12], sugarcane [13], and sorghum [14], highlighting its broad agricultural threats. Additionally, *F. verticillioides* can thrive under extreme environmental conditions, including high temperatures [15], and spreads efficiently through airborne conidia. These adaptive traits significantly increase its ecological and industrial threats.

Like many other plant-pathogenic fungi, *F. verticillioides* is capable of producing a wide spectrum of metabolites, including polyketides, non-ribosomal peptides, terpenoids, and quinones [16] that serve as phytotoxins [17] or signaling molecules [18]. Secondary metabolites usually have multifunctional roles during infection—not only as direct virulence factors that enable the fungus to manipulate the immune status, invasion, and microbiome of hosts, but also as agents that reshape host hormone signaling or prime fungal stress responses [19,20]. For example, the fungus produces fumonisins (FUMs), a class of water-soluble mycotoxins with a unique diester structure of polyhydroxy alcohols and tricarboxylic acids that show cross-species toxicity, such as causing loss of plant yield [21], lymphoid depletion in piglets [22], suppressing the immunity of hosts [23,24], and causing nephrotoxicity, pulmonary oedema, and carcinogenesis in humans [25,26,27]. The production of these molecules is tightly regulated by biosynthetic gene clusters (BGCs), including the well-characterized FUM cluster that is responsible for fumonisin biosynthesis. These reports highlight the current urgent need to dissect the metabolites produced by *F. verticillioides.*

In recent years, the crucial role of secreted proteins in mediating interactions between fungal pathogens and their hosts has been confirmed, such as modulating host immune responses, manipulating host cellular processes, and reprogramming metabolic pathways to favor pathogen survival and colonization [28]. The secreted proteins have been widely recognized as essential virulence factors in many pathogenic fungi [29]. For instance, in *Puccinia striiformis* (wheat stripe rust), the effector protein Pst18363 interacts with the wheat Nudix hydrolase TaNUD23, stabilizing the host protein and suppressing reactive oxygen species (ROS) accumulation, thereby facilitating infection [30]. *Candida albicans* can secrete proteins to assist its host immune escape, enhancing tissue adherence and invasive potential [5]. Similarly, the rice blast fungus *Magnaporthe oryzae* secretes the Alg3 protein to regulate its virulence and cell wall integrity [31]. A previous study showed that the striatin homologue Fsr1 of *F. verticillioides* forms an endomembrane-associated complex that regulates virulence, further supporting the central role of secreted proteins in pathogenicity [32]. These reports underscore the importance of secreted proteins in the pathogenicity of fungi, highlighting the importance of investigating secreted proteins in *F. verticillioides* to gain a deeper understanding of its pathogenic mechanisms and identify potential targets for disease control. However, the secretomic landscape of *F. verticillioides* remains to be revealed.

Over the past decade, fungal proteomics and metabolomics have been powerful tools for elucidating the molecular basis of pathogenicity. The secreted proteome offers novel insights into how fungi interact with their environment and host organisms [33]. Simultaneously, metabolomic profiling captures the metabolites of fungi that could be used to modulate host physiology, suppress host defenses, and other processes. The complete repertoire of secreted proteins and associated metabolites is collectively known as the fungal secretome. These omics strategies provide a global view of the secretome in the fungal infection process [34,35]. For instance, recent pan-secretome analysis has defined the core and host-specific secretomes of *F. proliferatum* [36], while other studies on *F. verticillioides* have identified 166 secreted proteins, particularly lignocellulolytic enzymes aimed at biomass degradation [37]. *F. verticillioides* has a great impact on both the agricultural industry and public health. However, the secretome of *F. verticillioides* remains to be revealed. To address this problem, we employed integrated proteomic and metabolomic approaches to profile the secretome of *F. verticillioides*, aiming to provide a theoretical basis for its biological characterization.

## 2. Materials and Methods

### 2.1. Fungal Strain

*F. verticillioides* strains were isolated from Yunnan Province, and deposited in the collection of Yunnan Key Laboratory of Veterinary Etiological Biology under accession number RMYN1. The fungus was preserved in 30% (*v*/*v*) glycerol solution and stored at −80 °C for long-term cryopreservation.

### 2.2. Growth Curve Measurement

*F. verticillioides* was cultivated on potato dextrose agar (PDA) medium at 25 °C in darkness. PDA, a nutrient-rich medium routinely used for *Fusarium* cultivation [38], was prepared using 12 g of commercial potato dextrose broth (Guangdong Huankai, Guangzhou, China) and 9 g of agar (Biofroxx, Einhausen, Germany) per 500 mL deionized water. Agar plugs (5 mm diameter) were transferred into 150 mL Erlenmeyer flasks containing 100 mL potato dextrose broth (PDB), which was prepared by dissolving 12 g of powder in 500 mL deionized water and autoclaving at 121 °C. Following inoculation, the flasks were incubated at 25 °C with shaking at 150 rpm for 7 days. At 24-h intervals, one flask was removed for analysis. Mycelia were harvested by centrifugation at 5000 rpm for 10 min, dried at 60 °C to constant weight, and biomass (g/day) was calculated.

In parallel, spore production was monitored daily. Aliquots from each flask were taken at 24-h intervals, diluted, and the number of spores was quantified using a hemocytometer (Shanghai Qiujing, Shanghai, China) under a microscope. A spore count growth curve was constructed with time as the x-axis and spore number as the y-axis.

### 2.3. Secretome Collection

Based on the above measurements, the culture supernatant corresponding to the rapid growth phase of *F. verticillioides* and the non-inoculated control medium were selected. This time point was specifically selected because it represents the peak of sporulation and high metabolic activity, ensuring the capture of an abundant secretome and sufficient metabolite production, while avoiding the release of intracellular proteins associated with fungal autolysis, which is often observed in the stationary phase. The fungal or non-inoculated control medium supernatant was collected on day 5. The samples were centrifuged at 10,000× *g* for 10 min, and the supernatant was carefully collected. The collected supernatant was then sent to a specialized service provider for proteomic and metabolomic sequencing.

### 2.4. Protein Digestion and LC-MS/MS Analysis

For protein digestion, samples were lyophilized and resuspended in SDT lysis buffer (4% SDS, 100 mM Tris-HCl), followed by the addition of TCEP/CAA mixture for reduction at 100 °C for 5 min. After cooling, proteins were concentrated using 10 kDa ultrafiltration tubes, and the samples were washed with UA buffer (8 M urea, 150 mM Tris-HCl, pH 8.0). Trypsin digestion was performed by incubating the samples with 6 µg trypsin in 40 µL of 50 mM NH_4_HCO_3_ at 37 °C for 16–18 h. After digestion, peptides were collected by centrifugation, desalted using C18 StageTips, and vacuum dried. The dried peptides were resuspended in 0.1% formic acid for concentration measurement and LC-MS analysis.

Peptides were analyzed using a Thermo Scientific Easy nLC 1200 system coupled with a Q-Exactive HF-X mass spectrometer. Chromatographic separation was performed on a C18 column with a linear gradient of acetonitrile (ACN) and 0.1% formic acid. The flow rate was 300 nL/min, and mass spectrometry was conducted in positive-ion mode. Full scans (350–1800 *m*/*z*) were followed by data-dependent acquisition (DDA) of the top 20 most intense ions. The MS1 resolution was 60,000 at *m*/*z* 200, while the MS2 resolution was 15,000 at *m*/*z* 200, with HCD activation and normalized collision energy of 28. The consistent presence of expected peaks and the clear separation of compounds further validate the reliability of the data, ensuring that the analysis accurately reflects the sample’s proteomic profile.

### 2.5. Metabolite Extraction and LC-MS/MS Analysis

The supernatant samples were thawed, vortexed, and extracted with 1 mL of precooled methanol–water (4:1, *v*/*v*). After sonication and centrifugation, the supernatant was collected and dried under vacuum. For analysis, the residue was reconstituted in methanol-water (1:1, *v*/*v*) and centrifuged. Chromatographic separation was performed on a SHIMADZU-LC30 UHPLC system (Shimadzu, Kyoto, Japan) with an ACQUITY UPLC^®^ HSS T3 column. The mobile phases were 0.1% formic acid in water (A) and acetonitrile (B), with a gradient increasing B from 0% to 100% over 12 min. Metabolites were analyzed using a QE Plus mass spectrometer (Thermo Scientific, Waltham, MA, USA) with HESI ionization in positive and negative ion modes. The mass spectrometer settings included a scan range of 75–1050 *m*/*z*, resolution of 70,000 @ *m*/*z* 200, and data-dependent acquisition (DDA) of the top 10 most intense ions for MS2 analysis. Data preprocessing was conducted using MSDIAL software (RIKEN, Yokohama, Japan), and metabolites were identified by matching *m*/*z* values and MS2 spectra with databases (HMDB, MassBank, GNPS) and an in-house library (BP-DB).

### 2.6. Bioinformatics Analysis

Given the exploratory, single-sample design, we report descriptive results without statistical inference. A blank-controlled background subtraction was applied using the non-inoculated medium. Features detected in the blank were removed, and interpretation focused on those uniquely detected in the inoculated culture supernatant. Venn diagrams were generated using the online tool jVENN (online version, http://jvenn.toulouse.inra.fr/app/example.html, accessed on 28 May 2025) to visualize the overlap of detected metabolites or proteins among different sample groups. The KOBAS 3.0 platform (http://kobas.cbi.pku.edu.cn, accessed on 28 May 2025) was used to perform KEGG pathway enrichment analysis of uniquely detected secretome proteins and metabolites. GO enrichment analysis of the identified proteins was performed using annotations from the UniProt and InterPro databases. Protein–protein interaction (PPI) networks were constructed using the STRING database (v11.5; https://string-db.org, accessed on 28 May 2025,). Protein IDs were mapped to STRING using the *F. verticillioides* genome as background, with interaction confidence scores set to ≥0.7 (high confidence). Visualization and clustering of network modules were performed using Cytoscape (v3.9.1).

## 3. Results

### 3.1. Growth Kinetics of F. verticillioides

**Figure 1:**
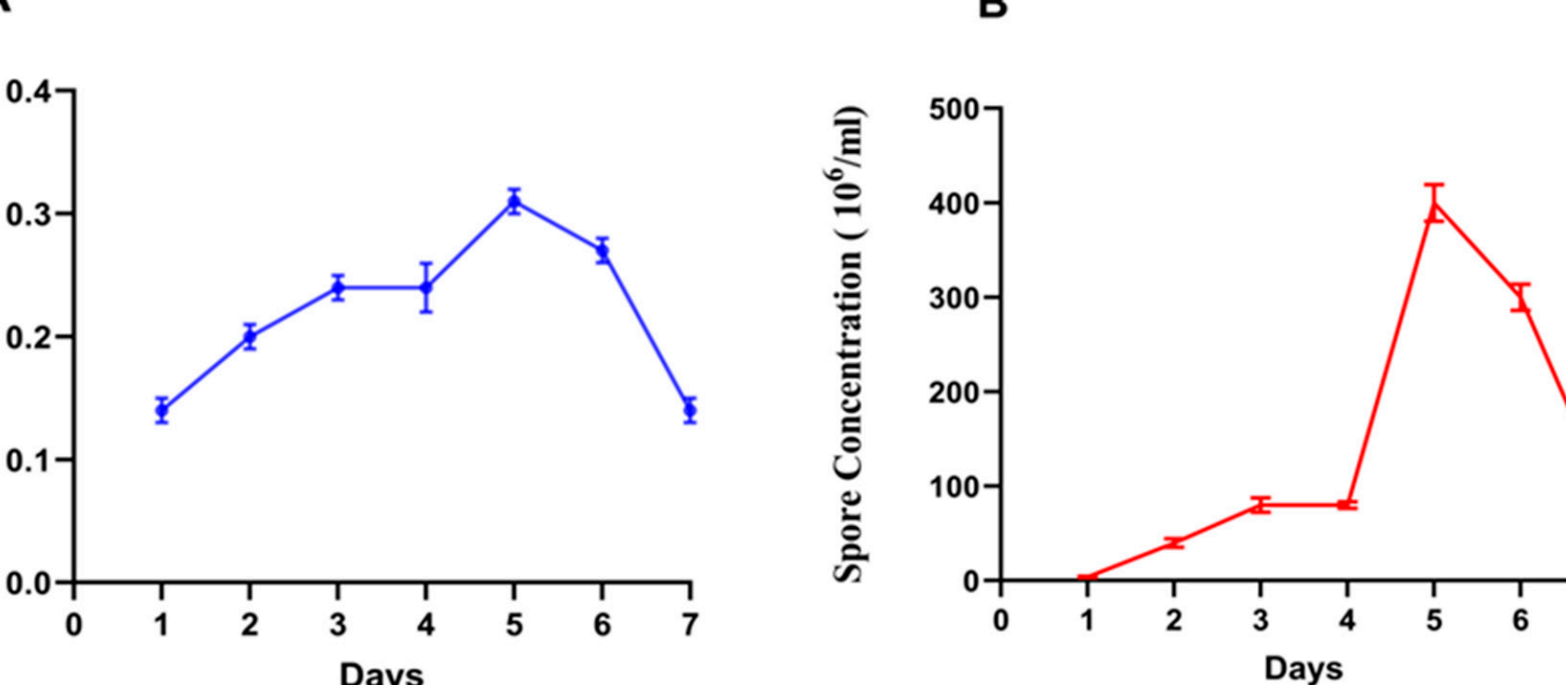
Growth kinetics of *F. verticillioides*. (**A**) The growth curve of *F. verticillioides*. (**B**) The spore count growth curve of *F. verticillioides* in liquid culture. Error bars indicate standard deviation (SD) (n = 3).

To characterize growth dynamics in liquid culture, we quantified the mycelial dry weight and conidiation over seven days. The growth rate increased from day 1 to day 5, peaking at 0.31 g/day, and then gradually declined (Figure 1A). Sporulation followed a similar trajectory, rising markedly after day 3 and reaching a maximum on day 5 (Figure 1B), indicating synchronized biomass accumulation and conidial production under the tested conditions.

### 3.2. Proteomic Profile of F. verticillioides Secretome

**Figure 2:**
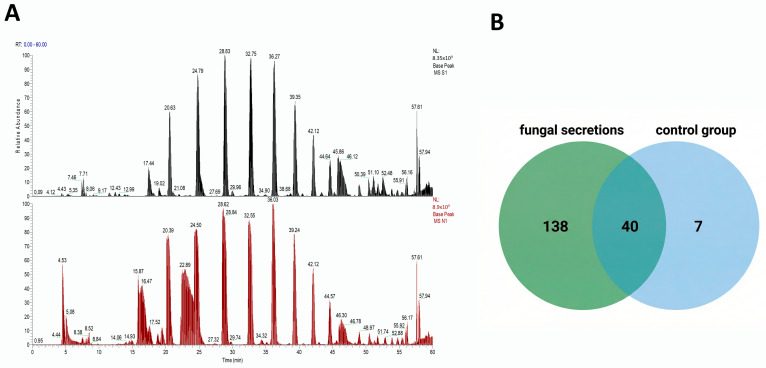
Proteomic profile of *F. verticillioides* secretions. (**A**) Base peak chromatogram (BPC) of proteomic analysis in positive ion mode. The chromatogram shows the intensity of the most abundant peaks (peptides) detected over time, confirming the high sensitivity and accuracy of the proteomic analysis. (**B**) Venn diagram of protein overlap between *F. verticillioides* secretions and the control group.

The secreted proteome was analyzed using liquid chromatography-mass spectrometry (LC-MS/MS). The base peak chromatogram (BPC) demonstrated clear and well-defined peaks, confirming the high sensitivity and accuracy of the sequencing process (Figure 2A). In the present study, proteomic analysis identified a total of 185 proteins in the culture medium supernatant of the fungal group, among which 138 proteins were exclusively found in the culture medium supernatant of the fungal group, 40 proteins were common to both fungal and control group, and 7 proteins were exclusively found in culture medium supernatant of control group (Figure 2B). As shown in Table S1, several fungal-specific proteins were identified, including chitinases such as W7M5U7, W7MWG4, and W7N4D9, as well as various hydrolases involved in polysaccharide degradation. Additionally, glycosidases like W7MNX3 and W7MVA were detected. Further analysis revealed the presence of CFEM domain-containing proteins, such as W7MAZ2, W7MJ13, W7LPA5, and W7LQW5. Proteins containing the WSC domain, including W7MWC8, W7M5U8, W7M103, W7MBN4, W7M168, and W7LZB2, were also identified. Additionally, carboxypeptidases such as W7MCV5 and W7MJ60 were present. The proteins containing the LysM domain, including W7N6T7 and W7MWW5, were also identified. Finally, we detected several uncharacterized proteins, including W7N0V7, W7LRU1, and W7LS08, which are predicted to be involved in murein transglycosylase activity. Further subcellular localization analysis of the unique secreted proteins revealed that 11 proteins were anchored components of membranes, and 13 proteins were intrinsic membrane components. Additionally, 19 proteins were localized to the extracellular region, eight to the fungal-type cell wall, and six to the cell surface.

### 3.3. Functional Enrichment and Protein–Protein Interaction (PPI) Network Analyses of Secreted Proteins

**Figure 3:**
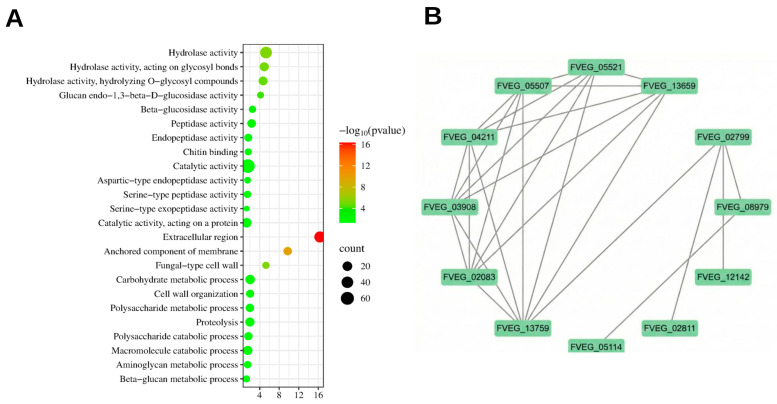
Proteomic profile of *F. verticillioides* secretions. (**A**) Gene ontology (GO) enrichment analysis of secreted proteins from *F. verticillioides*. This GO enrichment analysis highlights the functional categories of secreted proteins identified in *F. verticillioides* based on their biological processes, molecular functions, and cellular components. The x-axis represents the log-transformed *p*-values (−log10(*p*-value)), with higher values indicating greater statistical significance. The size of the circles correlates with the number of proteins involved in each category, with larger circles representing a greater number of proteins. (**B**) Protein–protein interaction (PPI) network of secreted proteins in *F. verticillioides.* This protein–protein interaction (PPI) network was constructed using the STRING database, illustrating the interactions between the secreted proteins identified in *F. verticillioides*. The nodes represent individual proteins, and the edges (colored lines) indicate potential interactions between them. The different colors of the edges represent various types of interactions based on the data sources used in the STRING database.

To better understand the characteristics of the secreted proteins (those unique proteins present in the inoculated culture) identified in the present study, gene ontology (GO) enrichment analysis was conducted. As shown in Figure 3A, a total of 24 significantly enriched GO terms were identified, and 3, 8, and 13 GO terms were classified into biological process, cellular component, and molecular function, respectively (Table S2). The GO enrichment analysis of biological processes showed that these secreted proteins are involved in several key cellular functions, including carbohydrate metabolism, polysaccharide metabolic processes, macromolecule catabolism, aminoglycan metabolism, and beta-glucan metabolism. Among the cellular components, the extracellular region, anchored components of the membrane, and fungal-type cell wall were significantly enriched with 36, 12, and 6 proteins, respectively. For molecular functions, hydrolase activity, glucan endo-1,3-beta-D-glucosidase activity, beta-glucosidase activity, peptidase activity, endopeptidase activity, chitin binding, catalytic activity, aspartic-type endopeptidase activity, serine-type peptidase activity, serine-type exopeptidase activity, and catalytic activity acting on a protein were significantly enriched. Furthermore, KEGG pathway enrichment analysis found that six proteins were enriched in “Starch and sucrose metabolism”, which was the sole significantly enriched metabolic pathway, highlighting its central role among the secreted proteins.

Also, we constructed a protein–protein interaction (PPI) network between the secreted proteins using the STRING database. As shown in Figure 3B, we identified 1,3-beta-glucanosyltransferase, glycosidases, and several hydrolases as key proteins involved in these interactions.

### 3.4. Metabolomic Profile of F. verticillioides Secretions

**Figure 4:**
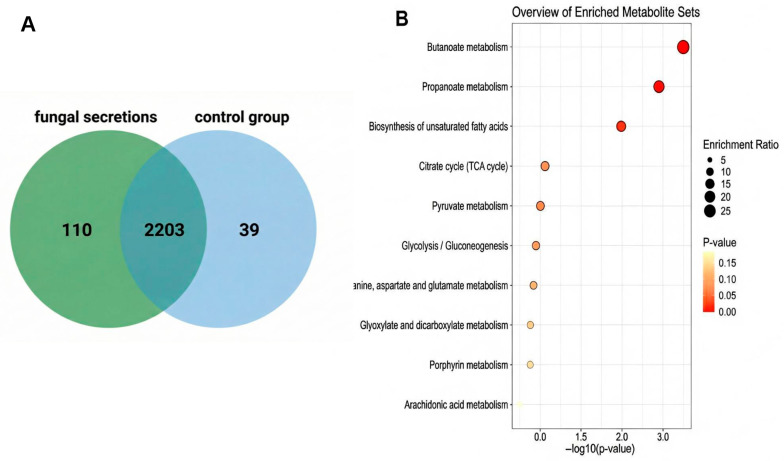
Metabolomic profile of *F. verticillioides* secretion. (**A**) Venn diagram of metabolites in *F. verticillioides* secretions. (**B**) KEGG enrichment analysis of secreted metabolites. This bar chart shows the enriched metabolite sets based on the KEGG pathway enrichment analysis of *F. verticillioides* secretions. The enrichment ratio represents the degree to which each pathway is enriched with metabolites from the fungal secretions.

To further investigate the metabolites secreted by *F. verticillioides*, we performed differential metabolomic analysis of culture media between the control group and the *F. verticillioides* group. The base peak chromatogram (BPC) analysis of the metabolomic data demonstrated clear separation of ion peaks corresponding to metabolites in both positive and negative ion modes, confirming the reliability and accuracy of the metabolomic profiling results (Figure S1). Metabolomic analysis of the *F. verticillioides* group showed that a total of 2352 metabolite features were identified. As shown in the Venn diagram (Figure 4A), 110 features were detected exclusively in the inoculated group and were absent in the control. Of these 110 unique features, 73 metabolites could be successfully annotated (identified by name). In total, 73 metabolites were deemed as the secreted metabolites for subsequent analysis. As shown in Table S3, these specifically secreted metabolites include fumonisins, fatty acids, and compounds such as benzophenones, morphinans, pyrroloindoles, ketones, naphthopyrans, carboxylic acids and derivatives, amino acids and derivatives, phthalides, medium-chain fatty acids, arylsulfates, and others.

### 3.5. KEGG Annotation and Enrichment Analysis of Secreted Metabolites

To evaluate the functional impacts of the annotated secreted metabolites, KEGG pathway enrichment analysis was conducted on the 73 identified compounds (Figure 4B). The enriched differential metabolism pathways mainly involved Butanoate metabolism, Propanoate metabolism, Citrate cycle, Pyruvate metabolism, Glycolysis/Gluconeogenesis, Glyoxylate and dicarboxylate metabolism, Porphyrin metabolism, Biosynthesis of unsaturated fatty acids, Arachidonic acid metabolism, etc. These pathways indicate its involvement in energy production and cellular function.

## 4. Discussion

*F. verticillioides* is a major limiting factor for maize production due to ear and stalk rot, and its contamination of seed with the carcinogenic mycotoxin fumonisin further exacerbates its agricultural and public health significance [39]. The dual impact of *F. verticillioides* on both food security and human health necessitates a comprehensive understanding of its virulence strategies. It is clear that the secreted compounds of fungi play important roles in pathogen virulence. However, the secretome of *F. verticillioides* remains to be revealed. We characterized the in vitro secretome of *F. verticillioides* grown in potato dextrose-based medium, and our interpretations are made within this defined culture context. We integrated proteomic and metabolomic analyses to systematically characterize the potential secreted compounds of *F. verticillioides*, revealing a diverse repertoire of hydrolytic enzymes and secondary metabolites closely linked to fungal virulence and host adaptation.

As shown in Table S1, 138 potential secreted proteins by *F. verticillioides*, glycosidases, and other carbohydrate-active enzymes (CAZymes) were prominently represented, particularly those targeting glycosyl bonds, β-glucans, and chitin, which are major components of plant cell walls. The enzymes of GH (glycoside hydrolase) families play key roles in cell wall degradation, nutrient acquisition, and modulation of host defenses [40]. For example, chitinases of the GH18 family are known to participate in tissue degradation, nutrient acquisition, pathogen invasion, and immune modulation [41]. Host plants (e.g., maize) frequently upregulate their own chitinase genes to reinforce the plant cell walls to resist *F. verticillioides* invasion [42]. However, to overcome chitin-induced plant immunity and establish a successful infection, many fungal pathogens secrete LysM domain-containing effector proteins during host colonization [43], highlighting a dynamic enzymatic arms race between hosts and pathogens. In our dataset, LysM domain-containing proteins (W7N6T7 and W7MWW5; Table S1) were identified. The detection of LysM domain proteins in the *F. verticillioides* secretome, therefore, highlights them as virulence-associated candidates that warrant functional validation in future host–pathogen interaction studies [44]. These proteins may enable *F. verticillioides* to evade host recognition and facilitate successful colonization. In the present study, we identified CFEM domain-containing proteins in the *F. verticillioides* supernatant of culture medium. The CFEM domain is known for its role in facilitating adhesion to host tissues and participating in host–pathogen interactions [45]. The presence of CFEM domain-containing proteins in the supernatant of the culture medium implies to us that these proteins may play roles in adhesion to host cells.

In this study, we found that WSC (wall stress component) domain-containing proteins from *F. verticillioides* were detected in the supernatant of culture medium. WSC domain-containing proteins (W7MWC8, W7M5U8, W7M103, W7MBN4, W7M168, and W7LZB2) are known for their carbohydrate-binding capacity, which can facilitate the anchoring of enzymes to cell wall components such as xylan and β-1,3-glucan, thereby improving enzymatic efficiency [46]. This synergy between adhesion proteins and cell wall-degrading enzymes likely facilitates host tissue colonization and enhances fungal virulence. Notably, the upregulation of endoglucanase and β-glucosidase genes in infected maize stalks is correlated with enhanced cellulose degradation and disease severity [47]. The enzymatic hydrolysis of cellulose into glucose not only provides essential carbon sources for fungal metabolism but also weakens the structural integrity of host tissues, promoting pathogen spread [34,48]. Consistent with this, our proteomic analysis detected several β-glucosidases (e.g., W7MNX3 and W7MVA0) and endoglucanase-like enzymes (e.g., W7MWT5 and W7LU28), supporting their role in *F. verticillioides* virulence (Table S1). In addition to glycosidases, we also identified carboxypeptidases (e.g., W7MCV5 and W7MJ60) in the secretome of *F. verticillioides* (Figure 2B, Table S1). Although not directly related to cellulose degradation, these proteases may contribute to the breakdown of peptide-rich substrates such as insect exoskeletons or environmental proteins, thereby broadening the ecological niche and adaptive capacity of the fungus [16]. Crucially, the reliability of our dataset is supported by its strong alignment with the conserved *Fusarium* ‘core secretome’ and the high abundance of key cell wall degrading enzymes (GH18), and effectors (LysM and CFEM) identified here mirror the findings of recent comparative secretome studies [36,37], confirming that our profile captures the authentic pathogenic machinery of the fungus.

The GO enrichment analysis reinforced these observations by highlighting significant enrichments of biological processes such as carbohydrate metabolism and polysaccharide metabolism, suggesting active polysaccharide degradation processes during *F. verticillioides* infection (Figure 3A). Hydrolase activities, especially glycosyl bond hydrolysis, further supported the enzymatic strategies employed by *F. verticillioides* to degrade plant structural polysaccharides. In addition, the “Starch and sucrose metabolism” pathway was significantly enriched by the potential secreted proteins of *F. verticillioides*, underscoring that growth on a potato–dextrose-based substrate drives the intensive utilization of readily available starch- and sucrose-derived carbon. This finding aligns with previous findings that *Fusarium* species can strategically target starch and sucrose metabolic pathways to efficiently exploit host nutrients [33].

The protein–protein interaction (PPI) network analysis provided insights into potential synergistic interactions among these enzymes. As shown in Figure 3B, key proteins, such as 1,3-beta-glucanosyltransferase and glycosidases, were centrally positioned within the interaction network, suggesting their critical roles in facilitating coordinated cell wall degradation and nutrient acquisition. These findings corroborate earlier studies demonstrating cooperative enzymatic activities among fungal hydrolases and glycosidases during infection processes.

In addition to hydrolytic enzymes, *F. verticillioides* is a prolific producer of secondary metabolites, including medium-chain fatty acids, porphyrin, sesquiterpenes, and fumonisins (Table S3). Our metabolomic data confirmed the production of fumonisins B1 to B4, with FB1 being the most abundant. This is consistent with findings that FB1 constitutes approximately 70% of the total fumonisin content [49]. FB1 is widely recognized as a potent mycotoxin that disrupts sphingolipid metabolism and is linked to severe health effects across species [16,50]. Therefore, the predominance of fumonisins in our dataset provides a mechanistic basis for host tissue damage and may facilitate colonization by weakening host defenses. The detection of medium-chain fatty acids (e.g., lauric acid) is also significant, as they play essential roles in fungal adaptation and survival. Previous studies suggest these compounds contribute significantly to fungal energy metabolism (via β-oxidation) and membrane integrity, which helps maintain redox balance and provides protection under hostile, host-induced oxidative stress conditions [51]. The presence of these fatty acids and porphyrin derivatives therefore suggests that *F. verticillioides* possesses adaptive mechanisms to oxidative stress during host colonization.

As shown in Figure 4B, KEGG enrichment analysis of metabolites highlighted several significantly enriched pathways, including the butanoate metabolism, propanoate metabolism, citrate cycle (TCA cycle), pyruvate metabolism, glycolysis/gluconeogenesis, glyoxylate and dicarboxylate metabolism, and arachidonic acid metabolism. The citrate cycle and pyruvate metabolism are essential pathways supplying energy and biosynthetic precursors, supporting fungal growth under nutrient-limited conditions encountered within host tissues. Similarly, glycolysis and gluconeogenesis play central roles in energy homeostasis and carbon assimilation, particularly important during fungal invasion and colonization stages. The enrichment of arachidonic acid metabolism is particularly intriguing, as arachidonic acid derivatives have been implicated in modulating host immune responses. Fumonisin B1 has been reported to interfere with arachidonic-acid-dependent signaling in mammalian cells [52], suggesting that *F. verticillioides* may use these metabolites to dampen host defenses and enhance infection success [41]. Beyond pathogenicity, this active arachidonic acid biosynthetic pathway represents a valuable genetic reservoir. Although the fungus is toxic, its efficient enzymes could be mined to engineer yield improvements in safer industrial oleaginous hosts [53].

Furthermore, sesquiterpenes serve as signaling molecules that regulate fungal development, helping the pathogen adapt to the host and establish infection [54]. Naphthoquinone pigments, with their antioxidant properties, scavenge reactive oxygen species (ROS) generated during host immune responses [55]. These candidate compounds may support the pathogen in overcoming host defenses, enhancing infection success. In addition, several classes of secondary metabolites observed here—including fumonisins, certain sesquiterpenes, and naphthoquinone-type compounds—belong to families of fungal phytotoxins with well-documented growth-inhibitory or necrotizing effects on plants, and some members of these families are being actively explored as leads for bioherbicide development [56,57]. Thus, defined fractions of the *F. verticillioides* secretome may not only contribute to pathogenesis but also serve as templates for environmentally friendly weed-control agents, pending detailed assessment of their selectivity and ecotoxicology.

Collectively, the findings of the present study highlight the multifaceted strategy employed by *F. verticillioides*, wherein secreted enzymes degrade structural barriers, secondary metabolites compromise host defenses, and metabolic flexibility supports survival in complex environments. Our multi-omics approach offers a comprehensive overview of these coordinated virulence mechanisms, revealing functional interconnections that were previously overlooked in studies focusing solely on fumonisin biosynthesis.

Despite the robustness of the proteomic and metabolomic data, a limitation of this study is that metabolomic technology cannot identify the origins of common metabolites. Therefore, we cannot draw the complete secreted metabolomic map of *F. verticillioides.*

## 5. Conclusions

By integrating proteomic and metabolomics technologies, this study provides a preliminary characterization of the complex secretome of *F. verticillioides*, identifying key proteins and metabolites that could be involved in fungal pathogenicity. We detected abundant cell wall-degrading enzymes together with fumonisins and other bioactive secondary metabolites, while enriched metabolic pathways point to adaptive strategies that support fungal survival and virulence in a starch-rich niche. The co-occurrence of degradative enzymes, mycotoxins, and redox-active metabolites suggests a multilayered virulence strategy in which enzymatic weakening of host tissues facilitates toxin penetration and persistence under host-induced stress. Overall, these findings enrich our understanding of *F. verticillioides* pathogenesis and provide a theoretical basis for designing targeted therapeutic strategies to combat *Fusarium* infection in crops.

## Data Availability

The metabolomics data have been deposited in the MetaboLights repository with the study identifier MTBLS13312. The mass spectrometry proteomics data have been deposited in the ProteomeXchange Consortium (https://proteomecentral.proteomexchange.org, accessed on 13 November 2025) via the iProX partner repository with the dataset identifier PXD070661.

## Supplementary Materials

The following supporting information can be downloaded at https://www.mdpi.com/article/10.3390/jof12010024/s1: Figure S1: Base Peak Chromatogram (BPC) of Metabolomic Analysis. Table S1: Details of Secreted Proteins. Table S2: Results of Secreted Protein enrichment analysis. Table S3: Results of Differential Metabolite Enrichment Analysis.
