# Supplementary figures and images for: Integrated Proteomic and Metabolomic Profiling of the Secretome of Fusarium verticillioides Reveals Candidate Associated Proteins and Secondary Metabolites
Source: J Fungi (Basel). 2025 Dec 27;12(1):24. doi: 10.3390/jof12010024 (PMC12842653; doi:10.3390/jof12010024)

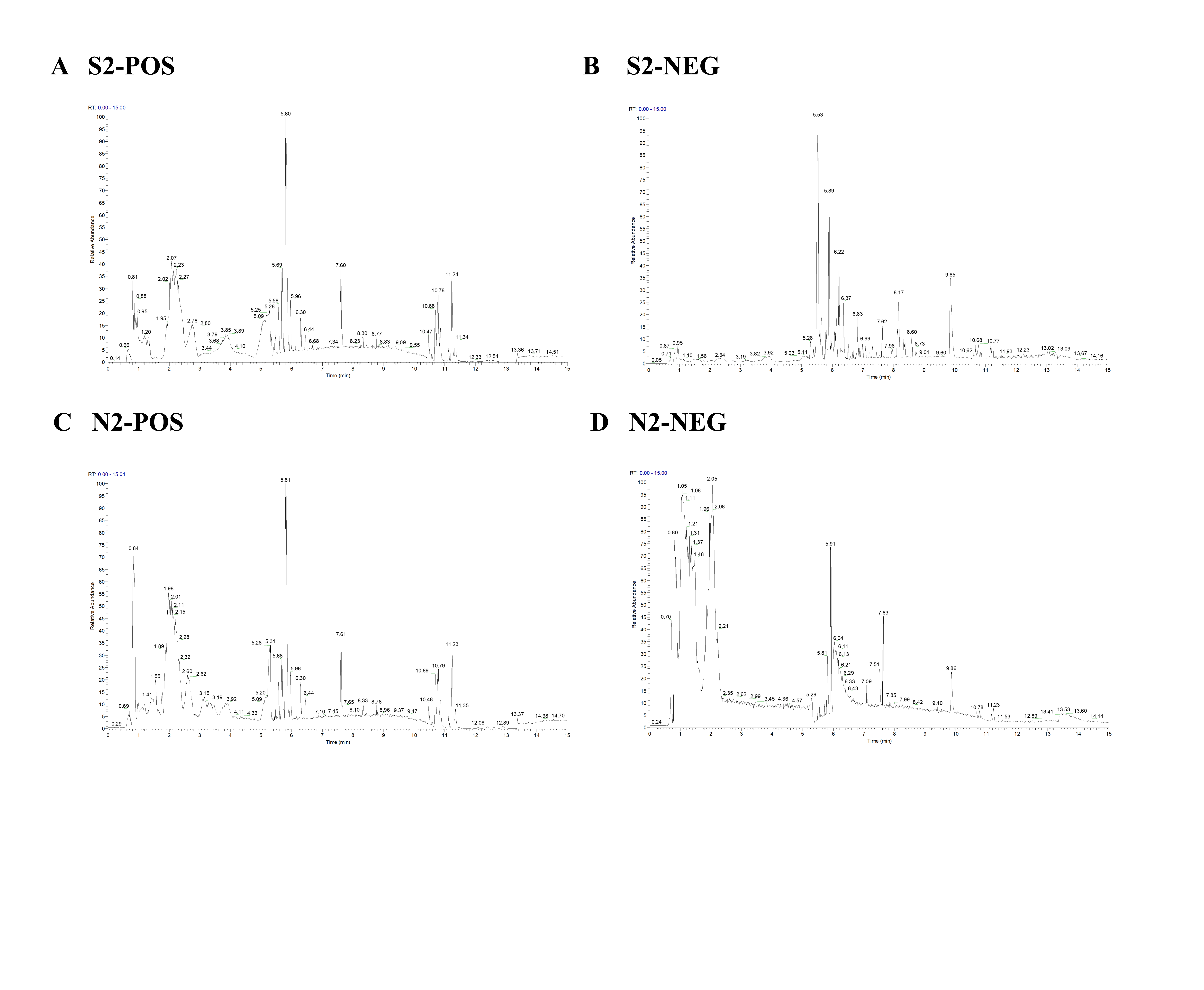

Supplement: Supplementary file 1 [file jof-12-00024-s001.zip › supplementary files/supplementary image and table/Figure S1.tif]
